# Supplementary material for: Effects of Propolis and Phenolic Acids on Triple-Negative Breast Cancer Cell Lines: Potential Involvement of Epigenetic Mechanisms
Source: Molecules. 2020 Mar 12;25(6):1289. doi: 10.3390/molecules25061289 (PMC7143942; doi:10.3390/molecules25061289)
Supplement: Supplementary file 1 [file molecules-25-01289-s001.pdf]

Article

Effects of Propolis and Phenolic Acids on Triple-Negative Breast Cancer Cell Lines: Potential Involvement of Epigenetic Mechanisms

João Henrique Maia Assumpção[1], Agnes Alessandra Sekijima Takeda[2], José Maurício Sforcin[1] and Cláudia Aparecida Rainho[1,*]

[1] Department of Chemical and Biological Sciences, Institute of Biosciences, São Paulo State University (UNESP), Botucatu, 18618-689, São Paulo, Brazil; joao.maia@unesp.br (J.H.M.A.); jose.m.sforcin@unesp.br (J.M.S.)

[2] Department of Biophysics and Pharmacology, Institute of Biosciences, São Paulo State University (UNESP), Botucatu, 18618-689, São Paulo, Brazil; agnes.takeda@unesp.br (A.A.S.T.)

* Correspondence: claudia.rainho@unesp.br; Tel.: +55-014-3880-0368

Supplementary figure S1

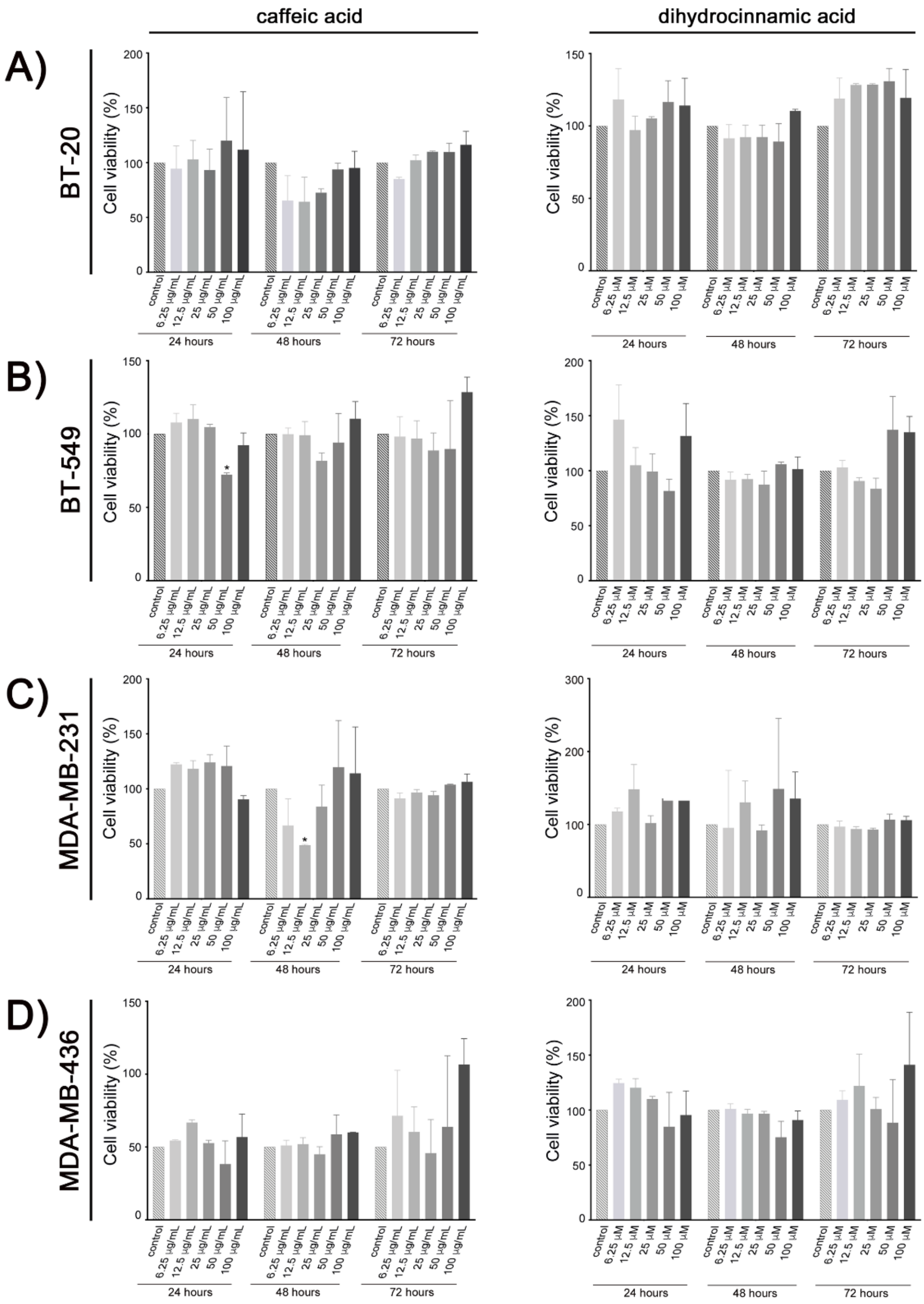

Figure 1. Cell viability analysis after in vitro treatment with caffeic and dihydrocinnamic acids of triple-negative breast cancer cell lines: BT-20 (A), BT-549 (B), MDA-MB-231 (C), MDA-MB-436 (D). Data represent means and standard deviation of three independent experiments. * p<0,05 in comparison with the untreated controls in the respective period of exposure (24, 48 or 71 h).

Supplementary figure S2

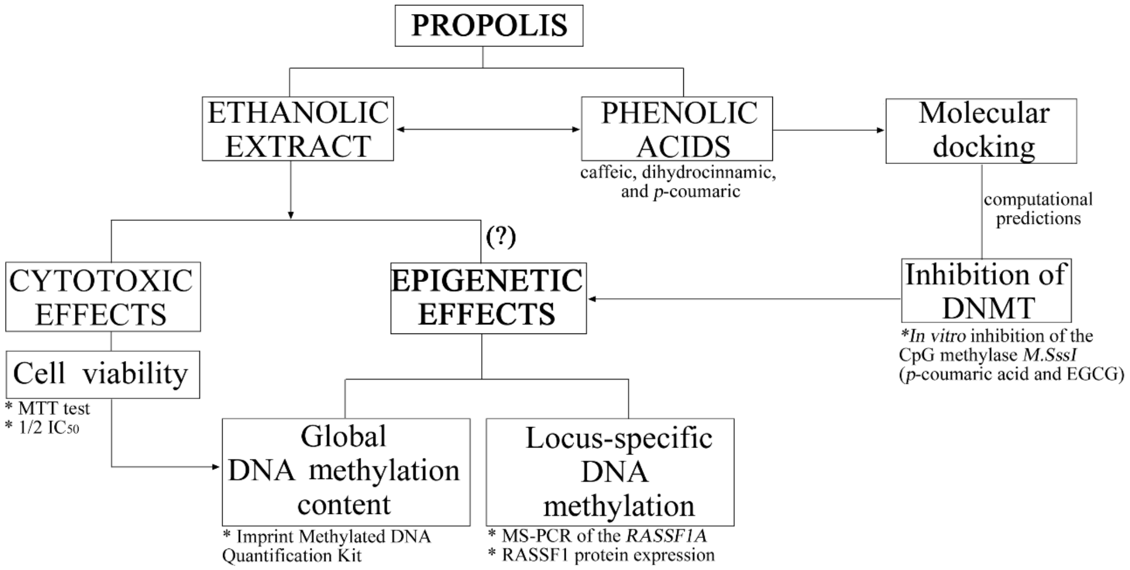

Figure 2. Study workflow. Three phenolic acids present in the EEP and four triple-negative breast cancer cell lines (BT-20, BT-549, MDA-MB-231, and MDA-MB-436) were selected for the present study. At first, the MTT test was used to evaluate the cell viability and determine the IC_{50} after in vitro exposure to the EEP and phenolic acids. The putative global hypomethylating effect of EEP was evaluated after 96 h of exposure at a dose of ½ IC*50* in the four cell lines. In parallel, computational docking predictions investigate the potential of the phenolic acids to interact with the catalytic domain of DNMT1, using the co-crystallography of DNMT1-SAH as well as the docking of EGCG as references. The in vitro inhibition of the CpG methylase *M.SssI* was used to validate the docking predictions. Methylation-Specific Polymerase Chain Reaction (MS-PCR) was used to analyze if treatment with EEP, p-coumaric acid and EGCG could revert the aberrant DNA hypermethylation of BT-549 cells. The *RASSF1* locus was the target region selected for this analysis.
